# Supplementary material for: Application of 3D MAPs pipeline identifies the morphological sequence chondrocytes undergo and the regulatory role of GDF5 in this process
Source: Nat Commun. 2021 Sep 10;12:5363. doi: 10.1038/s41467-021-25714-0 (PMC8433335; doi:10.1038/s41467-021-25714-0)
Supplement: Supplementary file 1 — Supplementary Information [file 41467_2021_25714_MOESM1_ESM.pdf]

Supplementary Information

Supplementary Methods

Data acquisition and segmentation

For tissue clearing, we used the PACT-deCAL technique [1, 2], which was found to allow nuclear labeling while preserving endogenous tissue fluorescence. Next, E16.5 nucleus- and cell membrane-labeled growth plates from tibias and ulnas were imaged by light-sheet fluorescence microscopy (LSFM) (Supplementary Figure 1A). To reduce the file size and expedite image processing, acquired z-stacks included only regions with growth plate nuclei and cells and images were downsampled in XY to a resolution lower than the cell membrane thickness prior to segmentation.

The next challenge was to conduct accurate and time-efficient 3D segmentation of nuclei and cells from the light-sheet images. For that, we combined two open-source platforms to perform semi-automatic segmentations (SAS) (Supplementary Figure 1B). Nuclei and cells from surrounding tissues were manually excluded by masking using Microview 2.1.2 (GE Healthcare). Next, the cleaned raw images were automatically segmented using specially designed pipelines for the nuclei and cells in XPIWIT software [3, 4] (S3 and S4 data). Then, nuclei and cells were filtered based on volume, and cells were analyzed only if they had a matching nucleus smaller in volume than the cell itself, and they had a voxel overlap of at least 50% (S6 and S7 data). On average, this segmentation stage resulted in the correct identification of 50% of nuclei and 25% of cells from all growth plate samples; for example, 124,400 nuclei and 62,200 cells in the proximal tibial growth plate (Supplementary Figure 1C).

To evaluate segmentation quality, we calculated the Rand index (RI), aggregated Jaccard index (IoU) and Dice coefficient (DC) [5-9] on pairs of cells and nuclei that were segmented both manually and automatically (Supplementary Figure 1D). The average IoU, RI, and DC were 0.83, 0.92, and 0.90 for cells and 0.71, 0.84, and 0.82 for nuclei, indicating segmentations of good quality [5, 6, 8]. To assess the impact of differences between the two segmentation methods, we computed both the percentage (%) deviation of several morphological characteristics (volume, surface area, sphericity, PC1/2/3 coefficient and orientation) per growth plate zone (Supplementary Figure 2), as well as the Bland-Altman test of agreement [10] (Supplementary Figure 3). The average % deviation (segmentation

error) per zone for each of the cell measurements were less than the biological differences we observed, suggesting that the segmentation error was below the resolution of the analyzed phenomena. The Bland Altman plots showed similar trends, but highlighted the spread of the segmentation errors from the mean and differences in the segmentation quality between zones. For example, across all measurements, the RZ and PZ cells had the lowest segmentation errors, while the hypertrophic zone cells had the largest errors.

To verify that the imaging angle does not introduce an artifact along the z axis, which would interfere with our ability to extract principal component 1, we analyzed the PC1 cell orientation of an ulna imaged at two orthogonal rotations along the P-D axis, such that the z axis was along either the dorsal-ventral or medial-lateral axis of the bone (Supplementary Figure 5). To show quantitatively that the PC1 cell orientations produced by orthogonal imaging were identical, we compared our results to the expected PC1 cell orientation errors (Supplementary Figure 2). The comparison revealed that differences were due to expected segmentation errors and not due to an imaging artifact, showing that we could accurately extract PC1 irrespective of the imaging angle.

Segmentation error calculation

To calculate segmentation error from all samples, we calculated the mean ratio of correctly segmented objects (nuclei or cells) out of the total object number in every image across all samples (n = 21 growth plates). For this, we counted the number of objects in a 2D optical section every 23 μm along the Z-axis, to avoid counting the same object twice. We then divided the number of automatically segmented objects in matching 2D sections to the number counted from the raw images to get the segmentation error for each image (S7 data). We considered a nucleus or cell to belong to a given 2D slice if its centroid was within 10 z planes (3.87 μm) from the given slice. We then averaged the errors within and across all samples (Supplementary Figure 1C) to calculate the mean error for nuclei and cells.

Accuracy of cell/nuclei segmentation

We manually extracted cell shape (n=500) and nucleus shape (n=405) from 3D binary images of the different zones of the growth plate. We computed the accuracy of the automatic segmentation using Jaccard index [5, 8], Rand index [9], and Dice coefficient [11]

between automated and manually extracted shapes. Manual segmentations were performed in MIB [12].

Jaccard index, also known as the Intersection-Over-Union (IoU), is the ratio of volume overlap between manual (ground truth) and automated (predicted segmentation) divided by the volume of union between the predicted segmentation and ground truth. First, we find the bounding box for each cell matching ground truth (G) to predicted segment (S), then counted the truth (signal) voxels and false (background) voxels using the equation:

(5)

$$\mathrm{IoU} = \frac{1}{2}\left[\frac{G == 1 \,\&\, S == 1}{G == 1 \mid S == 1} + \frac{G == 0 \,\&\, S == 0}{G == 0 \mid S == 0}\right]$$

IoU metric ranges from 0-1 with 1 indicating complete overlap and 0 indicating no overlap. Finally, we reported the histogram of IoU of all cells or nuclei and showed the frequency in percentage on the y-axis.

Rand index measures the similarity between two clustering agreements. If two clusters match perfectly, their rand index is 1. In image segmentation, it counts the number of true (signal) voxels and the number of false (background) voxels between G and S divided by the total volume of the bounding box, as in the equation:

(6)

$$\mathrm{RI} = \frac{(G == 1 \,\&\, S == 1) \;+\; (G == 0 \,\&\, S == 0)}{\text{total voxels in bounding box}}$$

Finally, we reported the histogram of RI of all cells or nuclei and showed the frequency in percentage on the y-axis.

Dice coefficient measures the overlap between two segmented images. It is the ratio of volume overlap between manual (ground truth) and automated (predicted segmentation) divided by the total number of voxels for both images combined. From the bounding box image of a cell or nucleus, it counts the number of signal voxels (truth) and background voxels (false) from the equation:

(7)

$$\text{Dice} = \frac{1}{2}\left[\frac{2(G == 1 \,\&\, S == 1)}{(G == 1) + (S == 1)} + \frac{2(G == 0 \,\&\, S == 0)}{(G == 0) + (S == 0)}\right]$$

Dice coefficient ranges from 0 to 1, where 1 denotes perfect overlap and 0 no overlap. Finally, we reported the histogram of Dice coefficients of all cells or nuclei and showed the frequency in percentage on the y-axis.

Accuracy of measured quantities between ground truth and segmented cells

We measured the accuracy in measured features (volume, surface area, sphericity, PC1 coefficient, PC2 coefficient, PC3 coefficient, deviation in PC1 orientation, deviation in PC2 orientation, and deviation in PC3 orientation) between ground truth (G) and automated segmented (S) shapes of cells per growth plate zone ($n_{rz} = 200$, $n_{pz} = 100$, $n_{phz} = 100$, $n_{hz} = 100$) in a similar manner to Stegmaier, Amat et al. (2016). We measured the absolute difference between G and S of each cell. For a feature F, if we measured Mg from G, and Ms from S, then the difference in percentage deviation between G and S is defined as

(8)

$$\%\ \text{deviation} = \frac{100|\text{Mg} - \text{Ms}|}{\text{Mg}}$$

For orientation features, the dot product between the Mg vector and Ms vector is computed and divided by 90 to ensure that the largest deviation occurs for perpendicular orientations. In this case, the percentage deviation is defined as:

(9)

$$\%\ \text{deviation} = \frac{100}{90}\left(M_g \cdot M_s\right)$$

where . is the dot product.

The % deviation is reported as a stacked histogram with the y-axis showing the frequency in percentage. The mean % deviation between G and S for each zone is given in the figure legends.

Bland-Altman plot (difference plot)

We examined the agreement between features computed by two different methods, namely manual segmentation (ground truth, G) and automated segmentation (S), using Bland-Altman plots [10], which detects differences such as fixed bias or possible outliers. For a given feature, if Mg is a measure from G and Ms from S, then the mean of Ms and Mg is plotted on the X-axis and the difference of Ms and Mg is plotted on the Y-axis. The 95% limit of agreement, which is 1.96 times the standard deviation of differences, is shown with dashed horizontal lines in the plots.

Quantification of nuclear pSmad 1/5/9

To quantify nuclear pSmad 1/5/9 in the growth plates of control and *Gdf5* KO mice, we used stained cryosections of proximal tibia. First, we performed masking of the growth plate, so the ROI consisted of only the proximal tibia growth plate. The hypertrophic zone was not included because it is shared with the distal tibia growth plate in the *Gdf5* KO mice. In Fiji [13], Gaussian blur (radius = 2) was performed on both the pSmad and DAPI channel. To count the number of nuclei in the entire ROI, the function "Find Maxima" was used on the DAPI channel, and the coordinates of all nuclei were exported into an Excel sheet. Using 3D ROI manager [14], nuclear volumes were measured across several regions, creating a volume filter of 10 - 75 μm^3. To count the number of cells with nuclear pSmad, a threshold of 17, 21, or 31 (depending on the staining experiment) was used to create a mask. The function Fill holes was used, and a label map was applied to the binary image with the Morpholib plugin [15] and was added to 3D ROI manager [14], where the volume for each pSmad-positive nucleus was calculated. Volumes within the nuclear volume filter of 10 - 75 μm^3 were saved in an Excel sheet. The ratio of nuclear pSmad/total nuclei was calculated for all sections analyzed. Two-way ANOVA (alpha = 0.05, statistical significance defined as $P<0.05$) was used to calculate the three highest ratios for each control and *Gdf5* KO group in each staining experiment (S8 data). Data are presented in a box and whisker plot.

Supplementary Table 1. List of features extracted by 3D MAPs.

Cell features	Nucleus features	Crossed features
Cells number	Nuclei number	Percentage of nuclei with matching cells
Occupation percentage	Occupation percentage	Nucleus/cell volume ratio (N/C ratio)
PC 1 coefficient	PC 1 coefficient	PC 1 alignment between nuclei and cells
PC 1 orientation	PC 1 orientation	PC 2 alignment between nuclei and cells
PC 2 coefficient	PC 2 coefficient	PC 3 alignment between nuclei and cells
PC 2 orientation	PC 2 orientation	Relative position of nucleus centroid from paired cell centroid
PC 3 coefficient	PC 3 coefficient	
PC 3 orientation	PC 3 orientation	
PC 2/PC 1 coefficient ratio	PC 2/PC 1 coefficient ratio	
PC 3/ PC 1 coefficient ratio	PC 3/ PC 1 coefficient ratio	
PC 3/PC 2 coefficient ratio	PC 3/PC 2 coefficient ratio	
Sphericity	Sphericity	
Surface area	Surface area	
Volume	Volume	
Volume$^{2/3}$/surface area	Volume$^{2/3}$/surface area	
Density	Density	
Delaunay density	Delaunay density	

Supplementary Table 2. List of primers used for animal genotyping

Primer name	Sequence
Cre	F-CCTGGAAAATGCTTCTGTCCGTTTGCC<br>R-GAGTTGATAGCTGGCTGGTGGCAGATG
GDF5creER	F-GTCAGTTGTGCGGGAGAAAGGG<br>R-ACCCCCTGGCCTAAAGACATTCC

Supplementary Table 3. Cell and nucleus volume ranges. To eliminate erroneous segmentations, we excluded from the analysis objects that had a volume outside of a given range. These ranges were set by manually selecting correctly segmented cells and nuclei from each zone and calculating their volumes in 3D morphology manager [14].

Cell volume range (μm^3)

Zone	Tibia WT	Tibia *Gdf5 KO*	Ulna WT
Resting	125 - 1400	125 - 1400	120 - 1500
Proliferative	300 - 1400	250 - 1400	120 - 2300
Prehypertrophic	1000 - 8000	1000 - 8000	1200 - 8000
Hypertrophic	1300 - 12400	1000 - 10000	1200 - 10000

Nucleus volume range (μm^3)

Zone	Tibia WT	Tibia *Gdf5 KO*	Ulna WT
Resting	125 - 550	100 - 850	100 - 1000
Proliferative	250 - 820	200 - 975	120 - 2000
Prehypertrophic	250 - 4600	200 - 2100	120 - 2500
Hypertrophic	300 - 4600	350 - 2100	300 - 2500

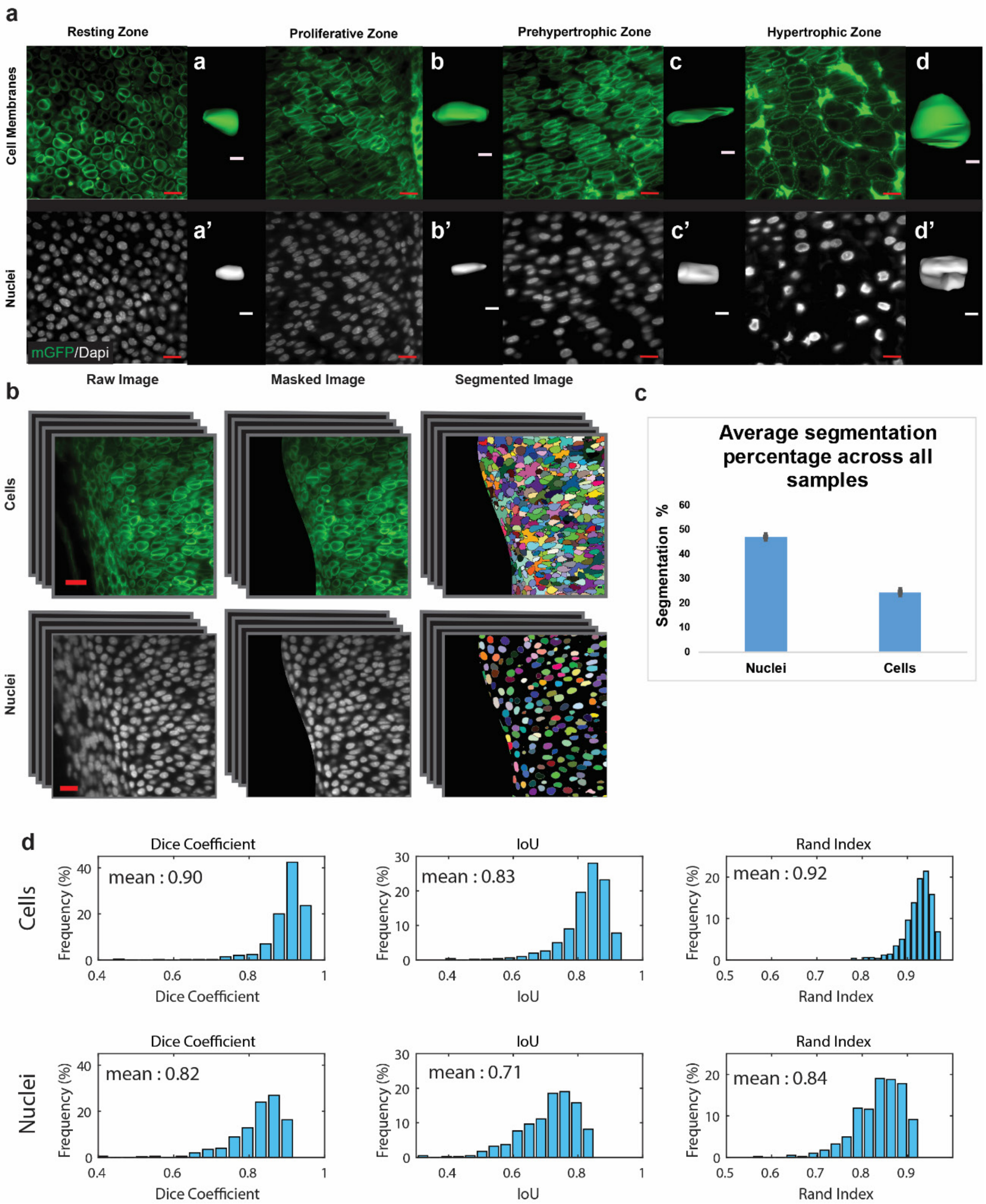
a
Resting Zone
Proliferative Zone
Prehypertrophic Zone
Hypertrophic Zone
Cell Membranes
Nuclei
a
b
c
d
a'
b'
c'
d'
mGFP/Dapi
b
Raw Image
Masked Image
Segmented Image
Cells
Nuclei
c
Average segmentation percentage across all samples
Segmentation %
60
50
40
30
20
10
0
Nuclei
Cells
d
Cells
Nuclei
Dice Coefficient
mean : 0.90
IoU
mean : 0.83
Rand Index
mean : 0.92
Dice Coefficient
mean : 0.82
IoU
mean : 0.71
Rand Index
mean : 0.84
Frequency (%)

Supplementary Figure 1. Data acquisition and segmentation. (**A**) Light-sheet images of cells and nuclei from the resting (RZ), proliferative (PZ), prehypertrophic (PHZ), and hypertrophic zones (HZ) were captured at 2.5X optical zoom with a 20X clarity lens of a Zeiss Light sheet Z.1 microscope. Cell membranes were endogenously labeled by using *mTmG:Col2a1-Cre* mice and nuclei were stained with DAPI. Scale bars: 20 µm. (a-d) 3D surface rendering of cells from the RZ, PZ, PHZ, and HZ. Scale bars: 5 µm. (a'-d') 3D surface rendering of nuclei from the RZ, PZ, PHZ, and HZ. Scale bars: 5 µm. n = 21 biologically independent samples examined over 12 independent experiments (**B**) Cells and nuclei undergo semi-automatic segmentation in Microview and XPIWIT. For both, the 3D raw image is masked, where the cells and nuclei not belonging to the growth plate are removed. Then, the masked image undergoes automatic segmentation for cells and nuclei with specially designed algorithms in XPIWIT to produce a 3D segmented image. Each color in the segmented image represents an individual object. Scale bars: 20 µm. (**C**) The average segmentation error was calculated for all growth plate samples by calculating the ratio of correctly segmented nuclei or cells out of the total nuclei in a z-stack. Error bar represents standard deviation from the mean across all samples (n=21 growth plates), resulting in on average 50% nuclei and 25% cells correctly segmented from each growth plate sample. (**D**) Segmentation quality was assessed using the Rand index (RI), aggregated Jaccard index (IoU) and Dice coefficient (DC) on cells or nuclei segmented both manually and automatically. The mean IoU, RI, and DC were 0.83, 0.92, and 0.90 for cells (n = 500) and 0.71, 0.84, and 0.82 for nuclei (n = 400).

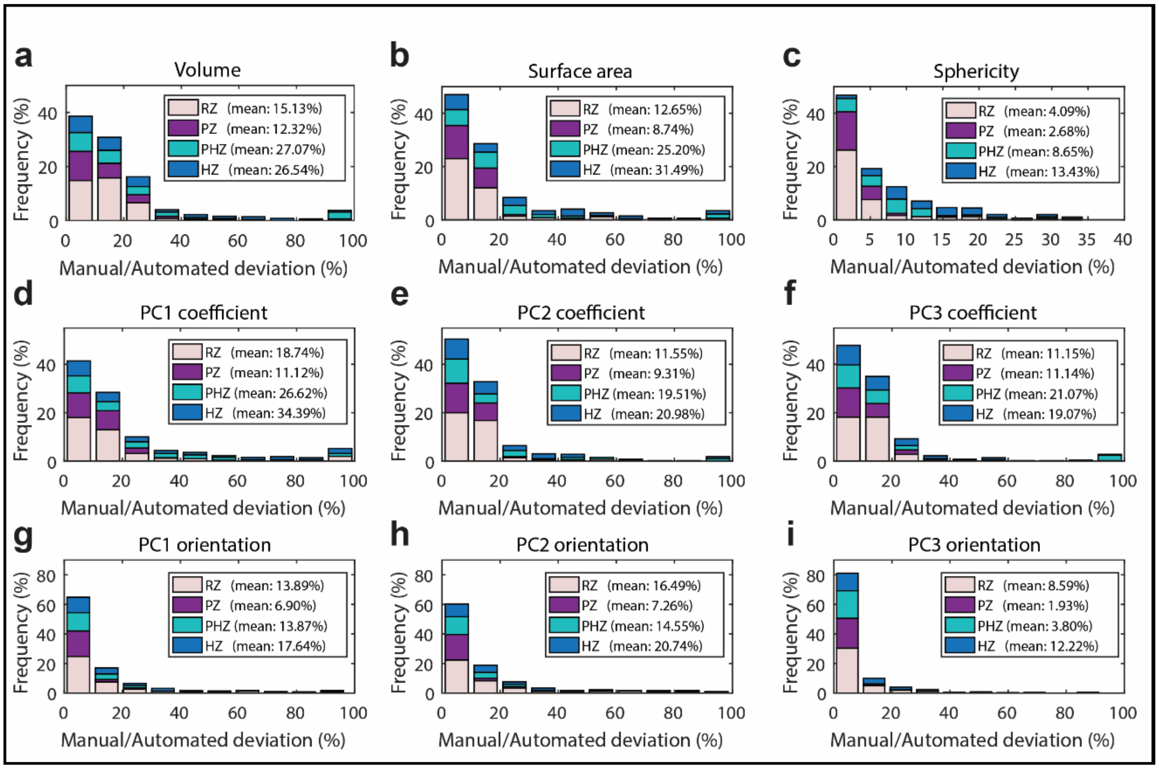

Supplementary Figure 2. Percentage deviation between manual and automated segmentations per zone. The percentage deviation was calculated for cell volume **(A)**, surface area **(B)**, sphericity **(C)**, PC1 coefficient **(D)**, PC2 coefficient **(E)**, PC3 coefficient **(F)**, PC1 orientation **(G)**, PC2 orientation **(H)**, and PC3 orientation **(I)**. $n_{rz} = 200$, $n_{pz} = 100$, $n_{phz} = 100$, $n_{hz} = 100$.

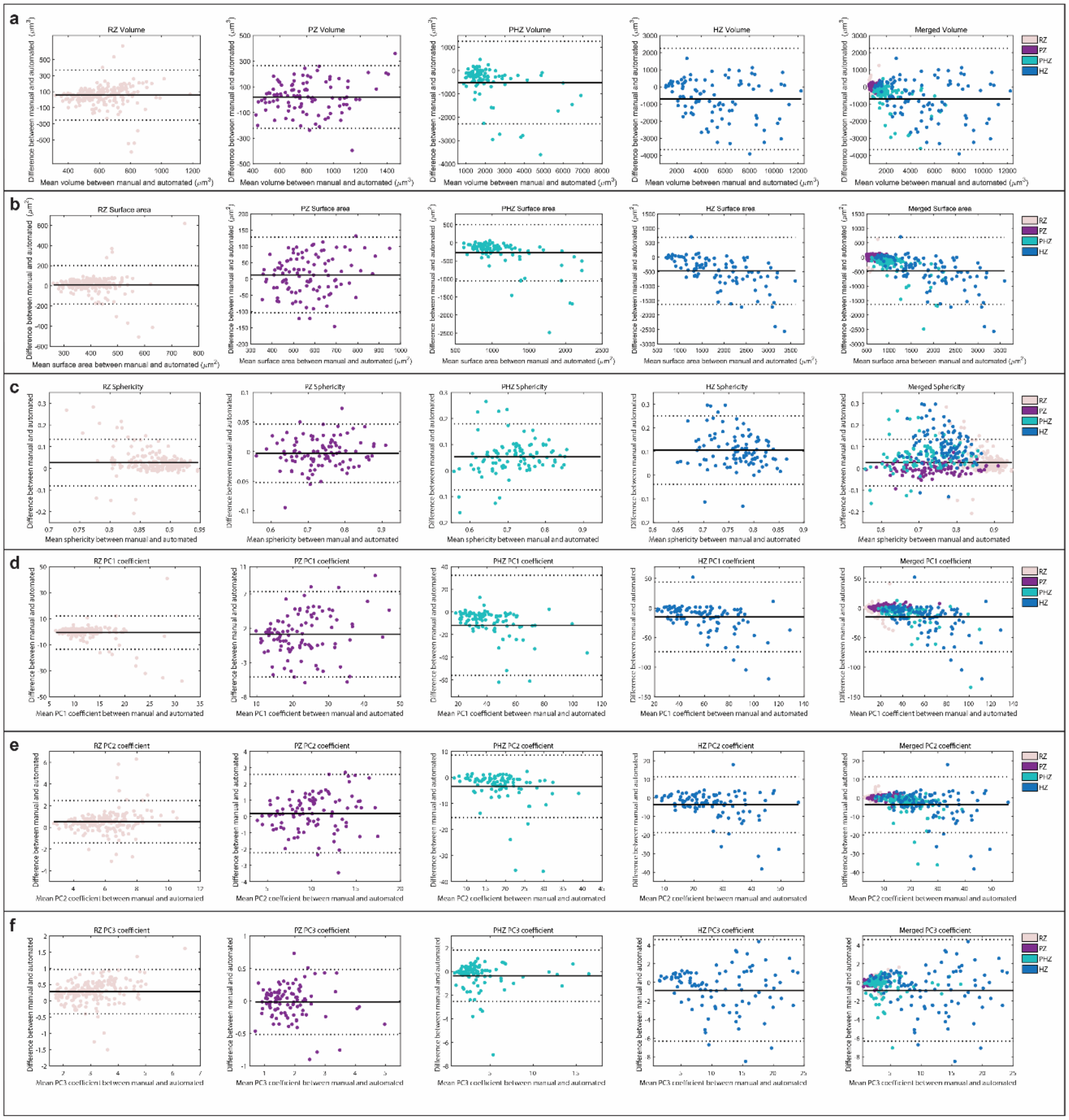

Supplementary Figure 3. Bland-Altman test of agreement between manual and automated segmentations per zone. The test was performed for cell volume (**A**), surface area (**B**), sphericity (**C**), PC1 coefficient (**D**), PC2 coefficient (**E**), and PC3 coefficient (**F**). $n_{rz} = 200$, $n_{pz} = 100$, $n_{phz} = 100$, $n_{hz} = 100$. The bold horizontal line on each graph denotes the mean of the data and the two dashed lines denote 2 σ, indicating that 95% of the data falls between them.

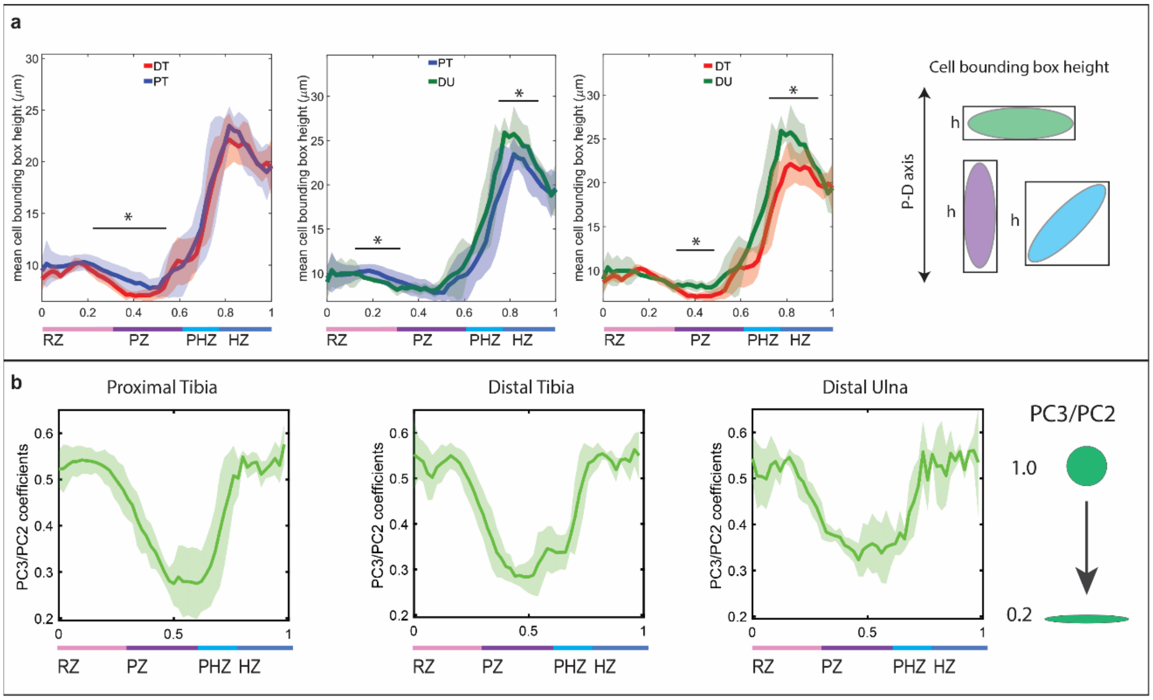

Supplementary Figure 4. Cell bounding box height and PC coefficient ratios along the P-D axis. (A) Spatial profiles of the mean cell bounding box height from the resting zone through to the end of the hypertrophic zone. Comparison between WT distal and proximal tibias shows that the mean cell height in the proximal tibia is significantly larger in the transition between the resting and proliferative zone ($p = 4.0e^{-03}$). Comparison between WT distal ulna and proximal tibia shows that the mean cell height in the proximal tibia is significantly larger in the resting zone ($p = 8.0e^{-03}$) and that of the distal ulna is significantly larger in the hypertrophic zone ($p = 2.0e^{-03}$). Comparison between WT distal ulna and distal tibia shows that the mean cell height in the distal ulna is significantly larger in the proliferative ($p = 1.8e^{-05}$) and hypertrophic zones ($p = 2.0e^{-03}$). On the right is an illustration depicting how the height of the cell bounding box is influenced by cell orientation. p-values were calculated by two tailed Student's *t*-test of means between samples (proximal and distal tibia, n = 5; distal ulna, n = 3). **(B)** The short-to-medium cell axis ratio (PC3/PC2) was plotted as a spatial profile along the differentiation axis. In all three growth plates, as cells differentiated from the RZ to the PZ, they decreased their PC3/PC2 ratio by half and then returned to the same ratio as the RZ when they differentiated to the HZ. Shaded regions denote standard deviations from the mean. Asterisks denote statistical significance.

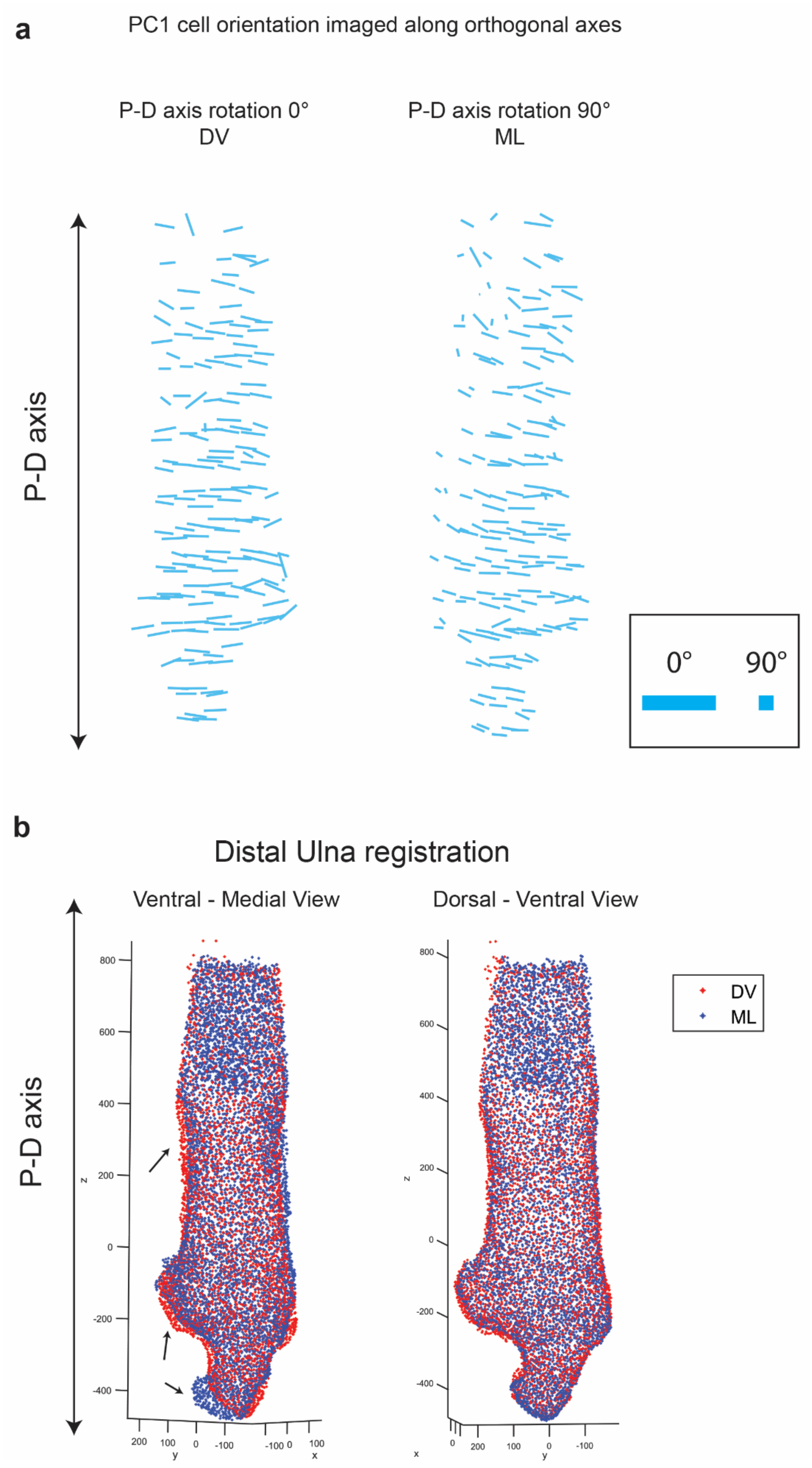
a
PC1 cell orientation imaged along orthogonal axes
P-D axis rotation 0°
DV
P-D axis rotation 90°
ML
P-D axis
0°
90°
b
Distal Ulna registration
Ventral - Medial View
Dorsal - Ventral View
P-D axis
DV
ML
800
600
400
200
0
-200
-400
z
200
100
0
-100
-100 0
100
y
x

Supplementary Figure 5. Comparison of PC1 cell orientation between two orthogonal imaging angles. (A) 3D morphology map of PC1 cell orientation of the same distal ulna sample imaged by light sheet microscopy at two orthogonal rotations (0˚, dorsal-ventral axis; 90˚, medial-lateral axis) shows high similarity between the two imaging rotations. (B) Two different views of the registered data (red, dorsal-ventral; blue, medial-lateral) show that they do not perfectly overlap (View 1). Black arrows indicate regions of low overlap that have the highest difference in PC1 cell orientation in panel A.

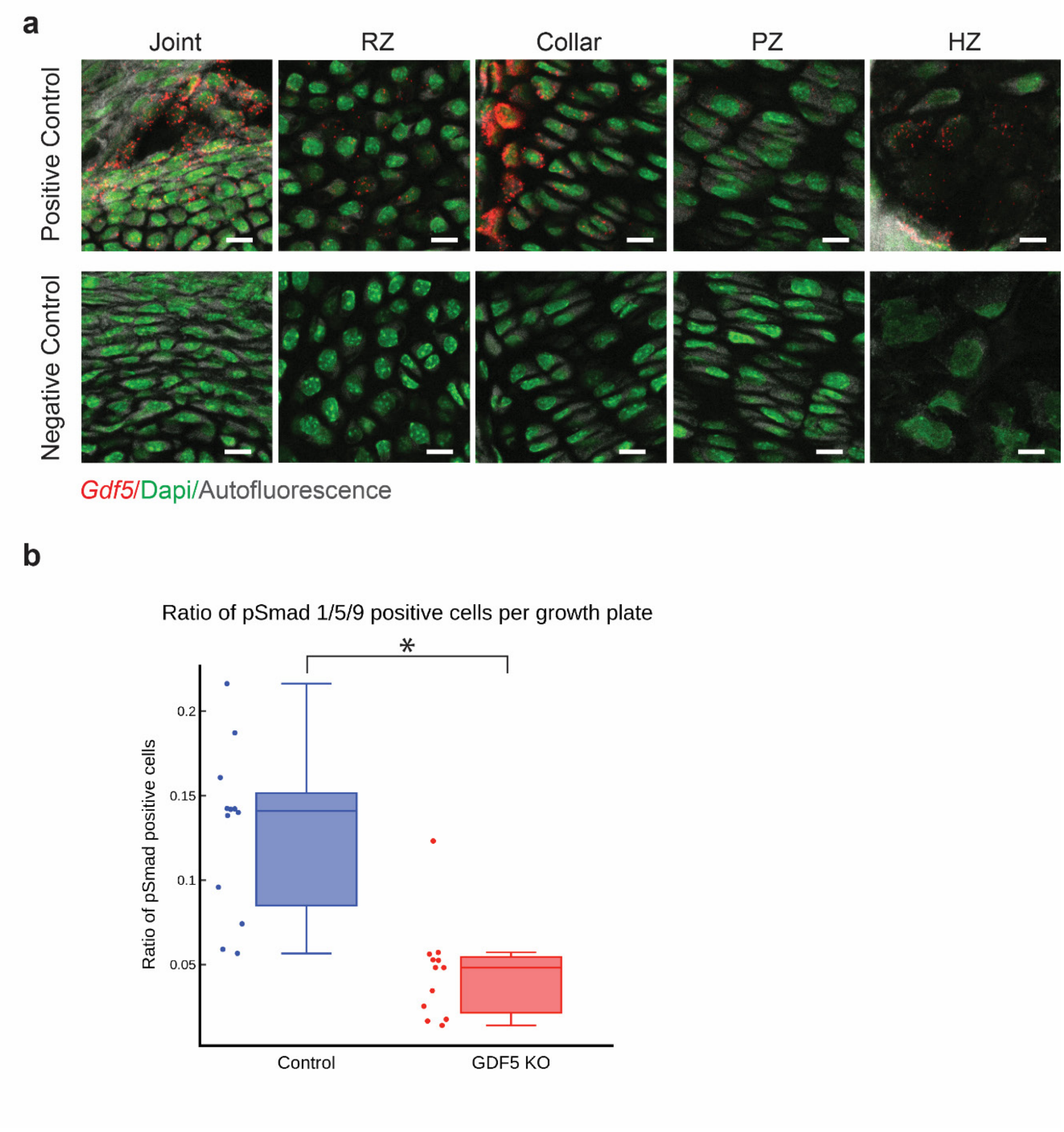

Supplementary Figure 6. *Gdf5* expression in the growth plate and quantification of SMAD 1/5/9 activity. (A) Positive and negative controls for HCR smFISH for *Gdf5* in the joint, resting zone, collar, proliferative zone, and hypertrophic zone of the proximal tibia. Scale bars: 10 µm. (n = 2 biologically independent samples examined over 2 independent experiments.) **(B)** Quantification of nuclear pSMAD 1/5/9 immunofluorescence from cryosections shows a reduction in the proximal tibia of *Gdf5* KO mice compared to controls (n =4). Asterisk denotes statistical significance of p = 1.8e^{-05} by two-way ANOVA. Box plots: horizontal line denotes median, lower and upper bounds of the box denotes 25th and 75th percentile respectively, the whiskers denote the minima and maxima of the data, points outside of the whisker range are outliers.

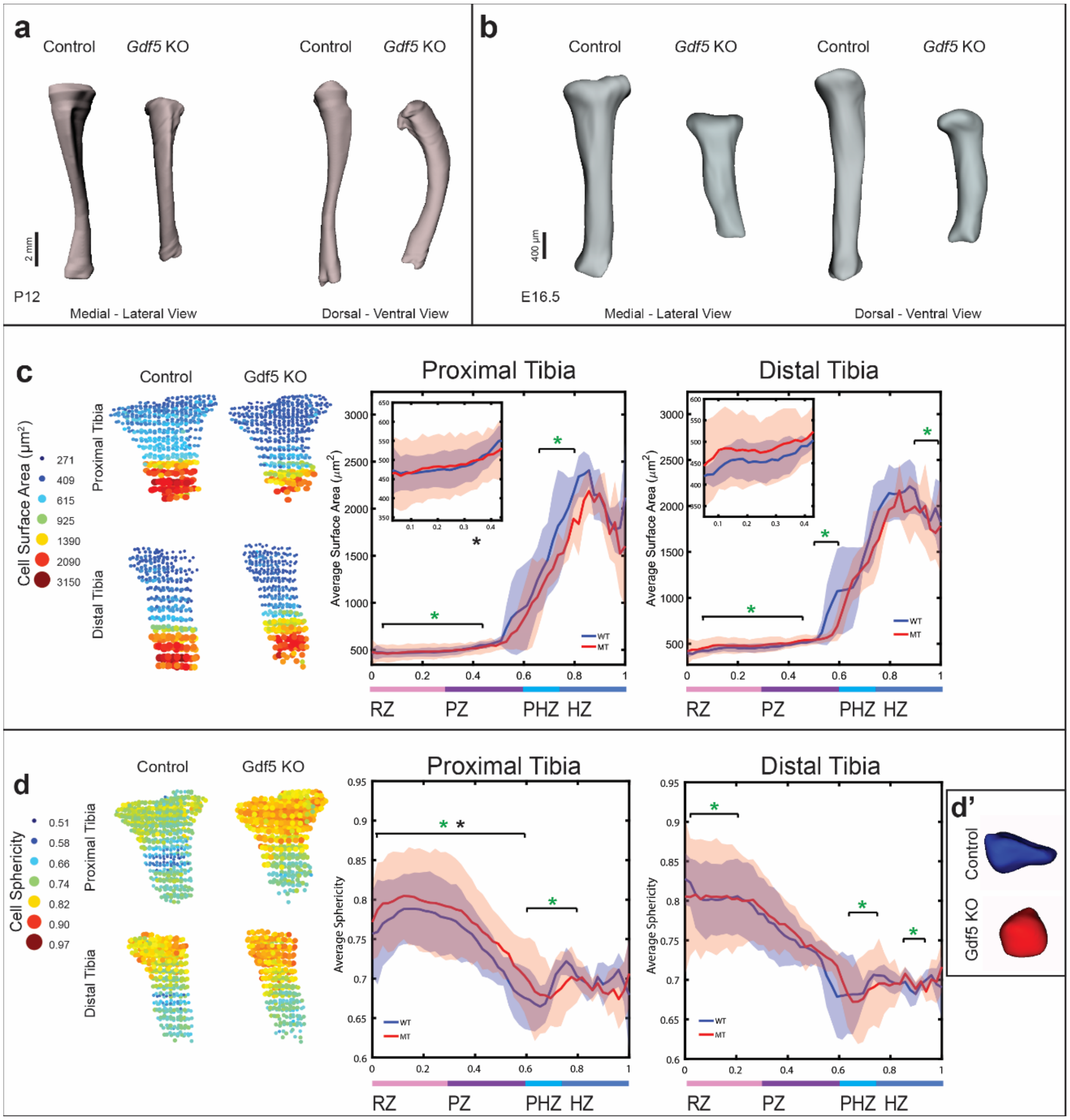

Supplementary Figure 7. GDF5 regulates surface area and sphericity of growth plate chondrocytes. (A) Surface renderings of micro-CT scans of P12 control and *Gdf5* KO tibias show that KO tibias are shorter than controls and have abnormal curvature. **(B)** Surface renderings of light-sheet scans of control and *Gdf5* KO tibias show that the morphological abnormalities are already present at E16.5. (**C** and **D**) Representative 3D maps of control and mutant samples as well as comparative spatial profiles show that *Gdf5* KO tibias display aberrant cell surface area and sphericity. In proximal (PT) and distal (DT) *Gdf5* KO tibial growth plates (**C**), the standard deviation of surface area was abnormally high in the RZ , PZ and DT HZ (RZ and PZ PT, $p = 3.4e^{-13}$, DT $p = 4.7e^{-13}$; DT HZ p = 0.025) and low in the PT PHZ (p = 0.005) and end of the DT PZ (p = 0.045). (**D**) Cells in the RZ and PZ of *Gdf5* KO growth plates had significantly higher mean sphericity in the PT (p = 0.021), indicating a loss of polarity and inability to flatten. This is also shown by cell surface renderings from the RZ of control and mutant growth plates (**D'**). The standard deviation of sphericity in mutants was abnormally high in the RZ, PZ, and PHZ of the PT and DT(RZ and PZ PT $p = 1.5e^{-07}$; PHZ PT $p = 2.1e^{-04}$; RZ DT $p = 6.8e^{-08}$; PHZ DT p = 0.003) and abnormally low in the DT HZ (p = 0.049). Shaded regions denote standard deviation from the mean. Black asterisks denote significant p-values calculated by two-tailed Student's *t*-test between means and green asterisks denotes p-values calculated by two-tailed Student's *t*-test between standard deviations (control, n = 5; mutant, n = 4). WT, control; MT, *Gdf5* KO.

Supplementary References

1. Treweek, J.B. *et al.* Whole-body tissue stabilization and selective extractions via tissue-hydrogel hybrids for high-resolution intact circuit mapping and phenotyping. *Nat. Protocols* **10**, 1860-1896 (2015).
2. Yang, B. *et al.* Single-Cell Phenotyping within Transparent Intact Tissue through Whole-Body Clearing. *Cell* **158**, 945-958 (2014).
3. Bartschat, A., Hübner, E., Reischl, M., Mikut, R. & Stegmaier, J. XPIWIT—an XML pipeline wrapper for the Insight Toolkit. *Bioinformatics* **32**, 315-317 (2015).
4. Stegmaier, J. *et al.* Fast Segmentation of Stained Nuclei in Terabyte-Scale, Time Resolved 3D Microscopy Image Stacks. *PLOS ONE* **9**, e90036 (2014).
5. Stegmaier, J. *et al.* Real-Time Three-Dimensional Cell Segmentation in Large-Scale Microscopy Data of Developing Embryos. *Developmental Cell* **36**, 225-240 (2016).
6. Stringer, C., Wang, T., Michaelos, M. & Pachitariu, M. Cellpose: a generalist algorithm for cellular segmentation. *Nature Methods* **18**, 100-106 (2021).
7. Zou, K.H. *et al.* Statistical validation of image segmentation quality based on a spatial overlap index. *Acad Radiol* **11**, 178-189 (2004).
8. Kumar, N. *et al.* A Dataset and a Technique for Generalized Nuclear Segmentation for Computational Pathology. *IEEE Transactions on Medical Imaging* **36**, 1550-1560 (2017).
9. Coelho, L.P., Shariff, A. & Murphy, R.F. NUCLEAR SEGMENTATION IN MICROSCOPE CELL IMAGES: A HAND-SEGMENTED DATASET AND COMPARISON OF ALGORITHMS. *Proc IEEE Int Symp Biomed Imaging* **5193098**, 518-521 (2009).

10. Martin Bland, J. & Altman, D.G. STATISTICAL METHODS FOR ASSESSING AGREEMENT BETWEEN TWO METHODS OF CLINICAL MEASUREMENT. *The Lancet* **327**, 307-310 (1986).
11. Dice, L.R. Measures of the Amount of Ecologic Association Between Species. *Ecology* **26**, 297-302 (1945).
12. Belevich, I., Joensuu, M., Kumar, D., Vihinen, H. & Jokitalo, E. Microscopy Image Browser: A Platform for Segmentation and Analysis of Multidimensional Datasets. *PLOS Biology* **14**, e1002340 (2016).
13. Schindelin, J. *et al.* Fiji: an open-source platform for biological-image analysis. *Nat Meth* **9**, 676-682 (2012).
14. Ollion, J., Cochennec, J., Loll, F., Escudé, C. & Boudier, T. TANGO: a generic tool for high-throughput 3D image analysis for studying nuclear organization. *Bioinformatics* **29**, 1840-1841 (2013).
15. Legland, D., Arganda-Carreras, I. & Andrey, P. MorphoLibJ: integrated library and plugins for mathematical morphology with ImageJ. *Bioinformatics* **32**, 3532-3534 (2016).
