# Supplementary material for: Application of 3D MAPs pipeline identifies the morphological sequence chondrocytes undergo and the regulatory role of GDF5 in this process
Source: Nat Commun. 2021 Sep 10;12:5363. doi: 10.1038/s41467-021-25714-0 (PMC8433335; doi:10.1038/s41467-021-25714-0)
Supplement: Supplementary file 3 — Reporting Summary [file 41467_2021_25714_MOESM3_ESM.pdf]

nature research

Corresponding author(s): Elazar Zelzer

Last updated by author(s): Jul 8, 2021

Reporting Summary

Nature Research wishes to improve the reproducibility of the work that we publish. This form provides structure for consistency and transparency in reporting. For further information on Nature Research policies, see our Editorial Policies and the Editorial Policy Checklist.

Statistics

For all statistical analyses, confirm that the following items are present in the figure legend, table legend, main text, or Methods section.

n/a	Confirmed	
☐	☒	The exact sample size (n) for each experimental group/condition, given as a discrete number and unit of measurement
☐	☒	A statement on whether measurements were taken from distinct samples or whether the same sample was measured repeatedly
☐	☒	The statistical test(s) used AND whether they are one- or two-sided<br>*Only common tests should be described solely by name; describe more complex techniques in the Methods section.*
☐	☒	A description of all covariates tested
☐	☒	A description of any assumptions or corrections, such as tests of normality and adjustment for multiple comparisons
☐	☒	A full description of the statistical parameters including central tendency (e.g. means) or other basic estimates (e.g. regression coefficient) AND variation (e.g. standard deviation) or associated estimates of uncertainty (e.g. confidence intervals)
☐	☒	For null hypothesis testing, the test statistic (e.g. F, t, r) with confidence intervals, effect sizes, degrees of freedom and P value noted<br>*Give P values as exact values whenever suitable.*
☐	☒	For Bayesian analysis, information on the choice of priors and Markov chain Monte Carlo settings
☐	☒	For hierarchical and complex designs, identification of the appropriate level for tests and full reporting of outcomes
☒	☐	Estimates of effect sizes (e.g. Cohen's d, Pearson's r), indicating how they were calculated

Our web collection on statistics for biologists contains articles on many of the points above.

Software and code

Policy information about availability of computer code

Data collection	Zen black (2014) software for Lightsheet microscopy (Zeiss) and Zen 2 blue (2014) for Confocal Microscopy (Zeiss) was used to collect the image data.

Data analysis	S1 data: Matlab (R2016b) script converts tif image file to a vff file to be used in Microview (2.5.0)<br><br>S2 data: Python(3.7.3) script converts xml contour file from Microview into 3D binary mask tif file<br><br>S3 data: Automatic 3D nuclear segmentation of Dapi intensity image in XPIWIT (1.0)<br><br>S4 data: Automatic 3D cellular segmentation of cell membrane intensity image in XPIWIT (1.0)<br><br>S5 data: Resaves labeled binary image to 12 unlabled binary images to avoid fusing of close objects in next stage<br><br>S6 data: creates heatmap of number of transcripts per cell in CellProfiler (3.1.9)<br><br>S7 data: uses Find Maxima to extract coordinates of nuclei every 60 slices in Fiji (2.1.0) then calculates segmentation error between automatic segmentation and GT in Matlab (R2016b)<br><br>S8 data: Quantification of nuclear pSmad1/5/9.

For manuscripts utilizing custom algorithms or software that are central to the research but not yet described in published literature, software must be made available to editors and reviewers. We strongly encourage code deposition in a community repository (e.g. GitHub). See the Nature Research guidelines for submitting code & software for further information.

Data

Policy information about availability of data

All manuscripts must include a data availability statement. This statement should provide the following information, where applicable:

- Accession codes, unique identifiers, or web links for publicly available datasets
- A list of figures that have associated raw data
- A description of any restrictions on data availability

The datasets generated during this study amounts to 21.5 Terabytes. We have uploaded one minimal sample dataset to Figshare, but the rest we prefer to make it available upon request. Below is the Data Availability statement in the manuscript.

The datasets generated and analysed during the current study are available from the corresponding author on reasonable request. Additionally, source data are provided within this manuscript and 3D MAPs sample data to test the codes are available on Figshare with the identifiers [https://doi.org/10.6084/m9.figshare.14903052.v1] and [https://doi.org/10.6084/m9.figshare.14932503.v1].

Field-specific reporting

Please select the one below that is the best fit for your research. If you are not sure, read the appropriate sections before making your selection.

☒ Life sciences ☐ Behavioural & social sciences ☐ Ecological, evolutionary & environmental sciences

For a reference copy of the document with all sections, see nature.com/documents/nr-reporting-summary-flat.pdf

Life sciences study design

All studies must disclose on these points even when the disclosure is negative.

Sample size	For 3D MAPs analysis we analyzed sample sizes n control=5 and n mutant = 4. Each dataset consisted between 30,000-60,000 cells, which was enough to show differences despite variations within and between samples. For immunohistochemistry experiments, we analyzed multiple sections from sample size of 4 control and mutants to overcome staining variabilities. This sample size was chosen because n= 3 is standard in the field so we used n=4 to be sure we had enough data to overcome staining variability and to show statistical significance between the control and mutants. For in situ experiments, we used sample size of 3, because the data was highly reproducible and with very little noise. For benchmarking analysis we chose n = 500 for cells and n = 400 for nuclei, 100 per zone except for rz cells where it was 200. It was shown in Stegmaier, Amat et al. 2016, that a sample size between 50-262 cells is sufficient for this segmentation error analysis.
Data exclusions	No data was excluded
Replication	The experiments were repeated at least three times, and the results were reproducible.
Randomization	Samples were allocated into control vs mutant groups based on their genotype. Covariates were controlled by analyzing at least three samples from different litters.
Blinding	The investigators were not blinded to group allocation. Since the imaging was performed identically between groups, and all of the segmentation was performed by algorithms, there was no need for blinding.

Reporting for specific materials, systems and methods

We require information from authors about some types of materials, experimental systems and methods used in many studies. Here, indicate whether each material, system or method listed is relevant to your study. If you are not sure if a list item applies to your research, read the appropriate section before selecting a response.

Materials & experimental systems

n/a	Involved in the study
☐	☒ Antibodies
☒	☐ Eukaryotic cell lines
☒	☐ Palaeontology and archaeology
☐	☒ Animals and other organisms
☒	☐ Human research participants
☒	☐ Clinical data
☒	☐ Dual use research of concern

Methods

n/a	Involved in the study
☒	☐ ChIP-seq
☒	☐ Flow cytometry
☒	☐ MRI-based neuroimaging

Antibodies

Antibodies used	Primary anti-pSMAD1/5/9 antibodies (CST-13820, Cell Signaling Technology)<br>Biotin-conjugated secondary antibodies (711-065-152, Jackson ImmunoResearch)<br>DAPI (D9542, Millipore Sigma)
Validation	The antibodies were validated by the respective companies.<br>Cell Signaling Technology: "To ensure product performance, we validate all of our antibodies, in-house, in multiple research applications."<br>Jackson ImmunoResearch: "Based on immunoelectrophoresis and/or ELISA, the antibody reacts with whole molecule rabbit IgG. It also reacts with the light chains of other rabbit immunoglobulins. No antibody was detected against non-immunoglobulin serum proteins. The antibody has been tested by ELISA and/or solid-phase adsorbed to ensure minimal cross-reaction with bovine, chicken, goat, guinea pig, syrian hamster, horse, human, mouse, rat and sheep serum proteins, but it may cross-react with immunoglobulins from other species."<br>Millipore Sigma: Based on a Quality test available on their website, signed by Anna Bendersky, Quality Control Manager Quallity Control, Israel, their product stains the nuclei of fixed HEK293 or equivalent cells.

Animals and other organisms

Policy information about studies involving animals; ARRIVE guidelines recommended for reporting animal research

Laboratory animals	mTmG:Col2a1-Cre:Gdf5-CreER control and mutant mice were used in this study. 8 week male and female mice homozygote for mTmG,Col2a1-Cre, and heterozygote for Gdf5-CreER were crossed and pregnant females were sacrificed at E16.5 or E18.5 for experiments, generating control and Gdf5 KO embryos. For histological analysis of columns, pups from litters were sacrificed at P6. We used both males and females in our growth plate analyses. Genotyping was performed by fluorescence or PCR. All animals used in this study had access to food and water ad libitum and were maintained under controlled humidity and temperature (45–65%, 22 ±2°C, respectively).
Wild animals	No wild animals were used in the study.
Field-collected samples	No field collected samples were used in the study.
Ethics oversight	All animal experiments were pre-approved by the Institutional Animal Care and Use Committee (IACUC) of the Weizmann Institute.

Note that full information on the approval of the study protocol must also be provided in the manuscript.
